# Ultra-Wide Band Non-reciprocity through Sequentially-Switched Delay Lines

**DOI:** 10.1038/srep40014

**Published:** 2017-01-06

**Authors:** Mathew M. Biedka, Rui Zhu, Qiang Mark Xu, Yuanxun Ethan Wang

**Affiliations:** 1 Department of Electrical Engineering, University of California, Los Angeles, 405 Hilgard Avenue, Los Angeles, CA 90095, USA

## Abstract

Achieving non-reciprocity through unconventional methods without the use of magnetic material has recently become a subject of great interest. Towards this goal a time switching strategy known as the Sequentially-Switched Delay Line (SSDL) is proposed. The essential SSDL configuration consists of six transmission lines of equal length, along with five switches. Each switch is turned on and off sequentially to distribute and route the propagating electromagnetic wave, allowing for simultaneous transmission and receiving of signals through the device. Preliminary experimental results with commercial off the shelf parts are presented which demonstrated non-reciprocal behavior with greater than 40 dB isolation from 200 KHz to 200 MHz. The theory and experimental results demonstrated that the SSDL concept may lead to future on-chip circulators over multi-octaves of frequency.

As the complexity of communications systems increases, the desire for simple and robust solutions to streamline the interactions between users also increases. In anticipation of the next generation of wireless communications, the ability to transmit and receive electromagnetic signals simultaneously has become a necessity. Circulators have been utilized extensively for this purpose yet they require the use of non-reciprocal, ferrite-based magnetic materials^1^,^2^. Such circulators are cavities that support non-reciprocal resonant modes, which lacks wideband performance and can not be easily incorporated into modern integrated circuits (ICs). On the other hand, active circulators have been realized based on the non-reciprocal transfer behavior of transistors. In comparison with traditional circulators, they offer small physical size and compatibility with IC technology^3^,^4^,^5^ at the price of limited noise and power performance. These shortcomings keep them from being utilized in systems that require wide dynamic ranges^6^,^7^,^8^. The realization of non-reciprocity without exploiting magnetic material properties has been introduced in a number of past works^9^,^10^,^11^,^12^,^13^,^14^,^15^,^16^,^17^,^18^,^19^,^20^,^21^. In these previous works, the dielectric property of a conventional transmission line was modulated in time and space to break the material property symmetry. Such concepts were proposed in the field of photonics^9^,^10^,^11^ and in acoustics^12^. For RF applications, metamaterial with active transistors were proposed^13^,^14^. Time-modulation architectures were also applied to parametrically coupled resonators^15^,^16^ and a staggered commutating switched capacitor device^17^ to achieve non-reciprocity over a very narrow bandwidth. Broadband non-reciprocity at RF has been demonstrated on a printed circuit board and on a monolithic microwave integrated circuit (MMIC) based on the concept of Time-Varying Transmission Line (TVTL)^18^,^19^,^20^,^21^. The lowest operating frequency of the device is dictated by the longest delay of the TVTL one can implement. This may result in a large area for on-chip integration of the device operating at low RF frequencies. The depth of isolation of the TVTL over a broad bandwidth is also limited by a SINC function, unless a more complex non-uniform modulation or balanced architecture is used^20^,^21^.

In order to achieve non-reciprocity on-chip that is needed for full-duplex operations without the lower limits of the operating frequency or the isolation depth, the Sequentially-Switched Delay Line (SSDL) concept is proposed. SSDL relies on a series of switching actions that are sequentially added to the paths of electromagnetic wave propagation to break the time reversal symmetry and to achieve non-reciprocity.

In a SSDL device, high speed Single Pole Double Throw (SPDT) switches are first used to break the signal apart in time-domain into many slices of equal length and then route these slices of signals into two separate paths alternatively. As long as the switch timing is syncronized with the delay of the wave propagation, the signal slices from those two different paths can be re-combined through SPDT switches at the designated port in a seamless fashion. Note that the sequential switching pattern allows the waves propagating toward different directions being re-constructed at different ports, the SSDL device thus operates like an asymmetric circulator^17^, which separates the simultaneously transmitting and receiving (STAR) waves into different circuit ports. As only true time delay and switching actions are involved, the non-reciprocal operation of SSDL is independent of frequency and can be practically implemented over an extremly wide bandwidth. It does not alter or reflect the transmitting or receiving waves, yet providing theoretically infinite isolation between the two. With the SSDL strategy, one can realize a truly passive RF circulator with high isolation that can fit in a chip the size of a few square millimeters.

Compared to other magnetless non-reciprocal schemes^15^,^16^,^17^,^18^,^19^,^20^,^21^, the advantage of the SSDL circulator is its potentially unlimited bandwidth even with a form factor that is suitable for on-chip integration. Nevertheless, the realization of high speed, low loss semiconductor switches yet with good power handling will be the primary challenge for practical development of such a device.

## Concept of Sequentially Switched Delay Line

**Figure 1:**
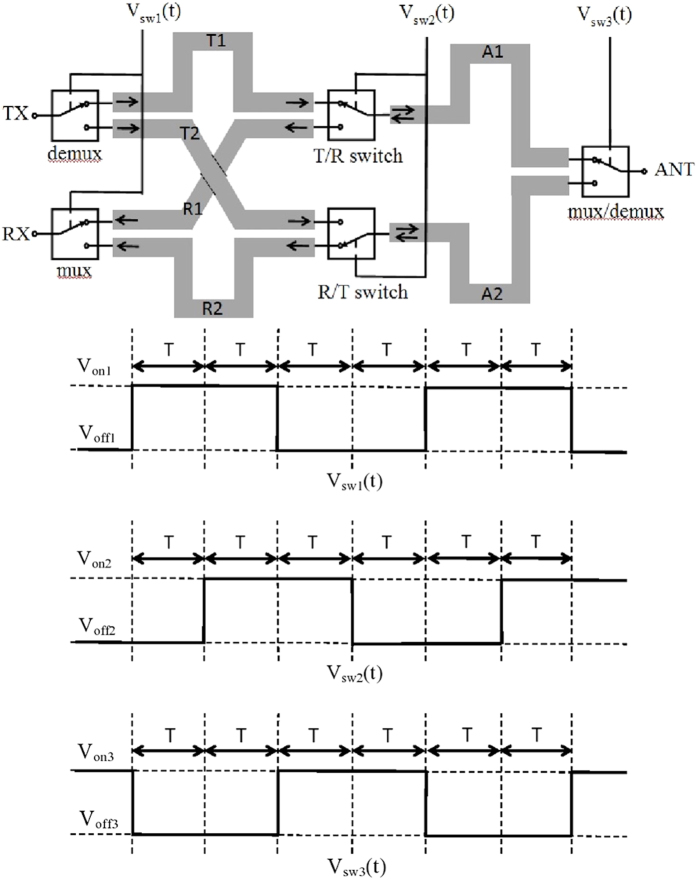
Schematic and control sequence of the circulator based on the SSDL concept. The design consists of 6 transmission lines and 5 switches. Transmitted signals are represented by the arrows pointing from left to right, and received signals are displayed as the arrows directed from the right to the left. The control voltage sequence allows for the simultaneous transmission and receiving of electromagnetic waves.

The concept of SSDL is illustrated in Fig. 1, which consists of six equal length transmission line segments with the delay time of *T* and five SPDT switches. There are three ports in the SSDL circulator, i.e. ports 1, 2, and 3 that are defined as the transmitting (TX), antenna (ANT), and receiving (RX) ports, respectively. Note that these three ports are not symmetrical, the port denomination indicates the preferred way of utilizing this device to provide the desired transmitter to receiver isolation in a single antenna wireless system. The operating principle is described as follows: The transmitted wave is split into two pulses through the TX demux switch. Each pulse has a duration of 2*T*. They follow a path of either TX->T1->A1->ANT or TX->T2->A2->ANT. The switches are turned on sequentially right before the wave arrives and turned off right after the wave departs. Finally the two pulses are combined at the ANT port through the ANT mux switch. For the received wave, the ANT port now acts as a demux switch. It divides the received wave into two 2T pulses in a similar fashion but at a time delay of 2T compared to that of the TX device. The received wave follows a path of either ANT->A2->R2->RX or ANT-A1->R1->RX, where the sequential switching pattern described previously allows for the propagation of the received waves. The T/R switch or the R/T switch is always turned to the opposing path the moment the wave in the previous path departs, so there is neither reflection nor alteration to the waveform.

In the case that the SSDL is operated in the non-denominated way, i.e., when a wave is launched into the RX port, it will encounter paths that are switched off, being bounced back and forth between the switches for a certain period of time and led to the TX port eventually. This is similar to what a standard circulator does except that the phase delays among the ports are not symmetrical. The details of such operation will be derived with the following time diagram analysis.

## Time Diagram Analysis of SSDL

**Figure 2:**
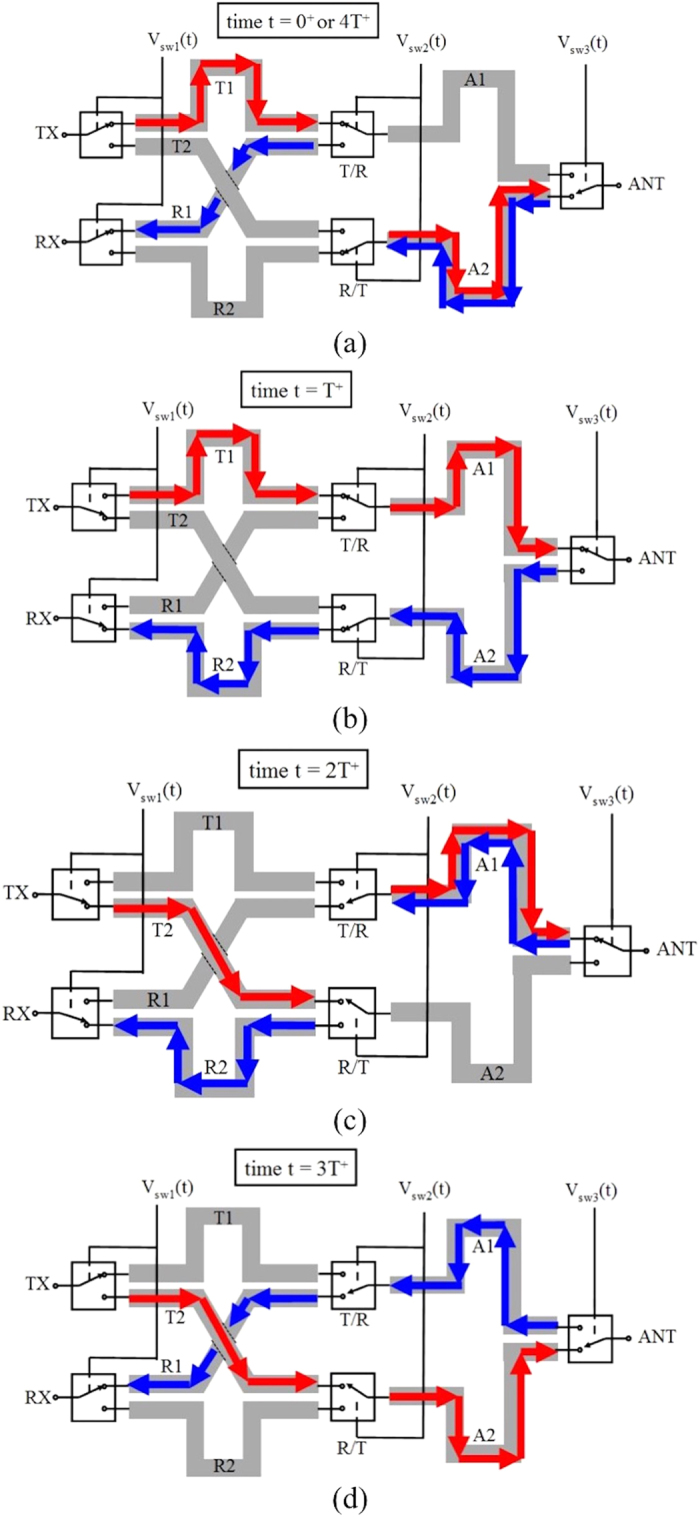
Timing diagram of the TX and RX waves during cyclic operations. The transmitted and received signals are represented by the colors red and blue, respectively. Individual figures (**a**) through (**d**) represent the positions of the switches at times 0^+^ to 3*T*^+^, respectively. Each instance in time is with respect to the T/R and R/T switches, and the (+) sign indicates that the switching action has just completed. The time progression shows that the transmitted and received signals propogate through the circulator without alteration or reflection, based upon the correct switching sequence. This allows the circulator to accommodate both the TX and RX waves without interferences. Note that progressing time to 4*T*^+^ causes the signals to return to their original positions in (**a**).

The dynamic process of wave propagation in SSDL can be well illustrated with the help of a time diagram. In general, the periodical switching actions in SSDL maintain a cyclic process with the exception of initialization in the first several cycles. When the SSDL device is operated in the designated way and synchronization between the wave propagation and the switching actions is enforced, it can be easily shown that only a time of 2*T* is needed for the device to enter into cyclic operations. The time diagram in Fig. 2 illustrates the process of wave propagation at the four switching moments of each cycle. The red arrows represent the transmitted wave and the blue arrows represent the received wave. Each switch is turned “on” to the correct transmission line for a time of 2*T*, and each instance in time is with respect to the switching action of the T/R and R/T switches. With this in mind, we will begin with Fig. 2a and explain how the signals propagate through the device. Figure 2a represents the location of the transmitted and received waves at time 0+. The (+) sign indicates that the devices have just completed their switching action. The T/R and R/T switches have just switched to the transmission lines T1 and R2, respectively. The TX and RX switches have been in their current state for a time of *T*^+^, and the first transmitted pulse leaves TX to populate T1. Also, the second received pulse is seen populating the R1 transmission line and arrives at the RX port. The ANT switch has also been on until the time *T*^+^, which allows for the first received pulse to begin populating A2. Also, the second transmitted pulse populates A2 and arrives at the ANT switch. The ANT switch will always have a switching action that opposes that of TX and RX. This is because the control signal for ANT is delayed by 2T relative to TX and RX. The transmitted and received signals that simultaneously populate A2 will not interfere with each other because they travel toward different directions. In Fig. 2b, we are looking at time *T*^+^, which is with respect to the switching action of the T/R and R/T switches. This means that the TX, RX and ANT switches flip from their previous positions in Fig. 2a, while the T/R and R/T switches remain in their respective positions. Now, the first transmitted pulse populates the entire 2*T* length of transmission line from T1 to A1. Furthermore, the first received pulse is allowed to fill the entire 2*T* length of transmission line from A2 to R2. It is worth noting that right before the switches flipped, the second transmitted pulse was captured at the ANT port, and the second received pulse arrived at the RX port. The switches are now in the correct positions so that the first transmitted pulse can be captured at the ANT port, and the first received wave can be collected at the RX port. As we progress to Fig. 2c, we are looking at the positions of the transmitted and received waves at time 2*T*^+^. The T/R and R/T switches have completed their switching action after being in their previous states for time 2*T*. The TX, RX, and ANT switches have remained in their respective positions. The first transmitted pulse populates A1 and can be seen entering the ANT port. At the same instant, the second received pulse leaves the ANT port and propagates along A1. The first received pulse can be seen entering the RX switch along transmission line R2, and the second transmitted pulse has left TX and populates T2. As time advances toward Fig. 2d, the transmitted and received waves are captured in the circulator at time 3*T*^+^. The T/R and R/T switches remain in their respective positions. The TX, RX, and ANT switches have completed their switching action after being in their previous states for time 2*T*. The second transmitted pulse now populates the full 2*T* length of transmission line from T2 to A2. Also, the second received pulse is allowed to populate the entire 2*T* length of transmission line from A1 to R1. If time progresses further to 4*T*^+^, the transmitted and received waves will return to their positions as seen in Fig. 2a.

**Figure 3:**
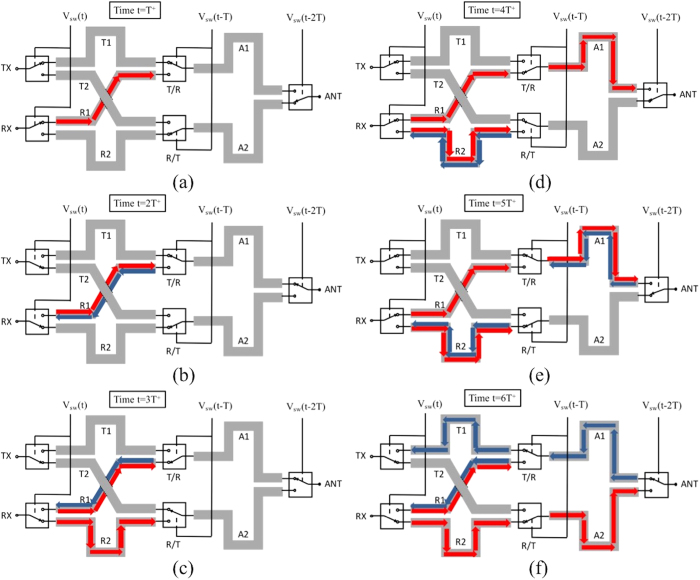
Time diagram for waves launched into the RX port during the initial state. The waves traveling toward the antenna side and the transceiver side are represented by the colors red and blue, respectively. Individual figures (**a**) through (**f**) represent the positions of the switches at times *T*^+^ to 6*T*^+^, respectively. Each instance in time is with respect to the T/R and R/T switches, and the (+) sign indicates that the switching action has just completed. The time progression shows that the input signals to the RX port propogate to the TX port in a period of 6*T*^+^, based upon the switching sequence identical to that in Fig. 2. Note that the device enters into cyclic operations after that.

When the device is operated in a non-dominated way or synchronization between the wave propagation and the switching actions is not met, it may take much longer time for the device to enter into cyclic operations. When waves are launched into the RX port, the time diagram in Fig. 3 shows the complete process of the wave propagation during the initial state before the device enters into cyclic operations.

**Figure 4:**
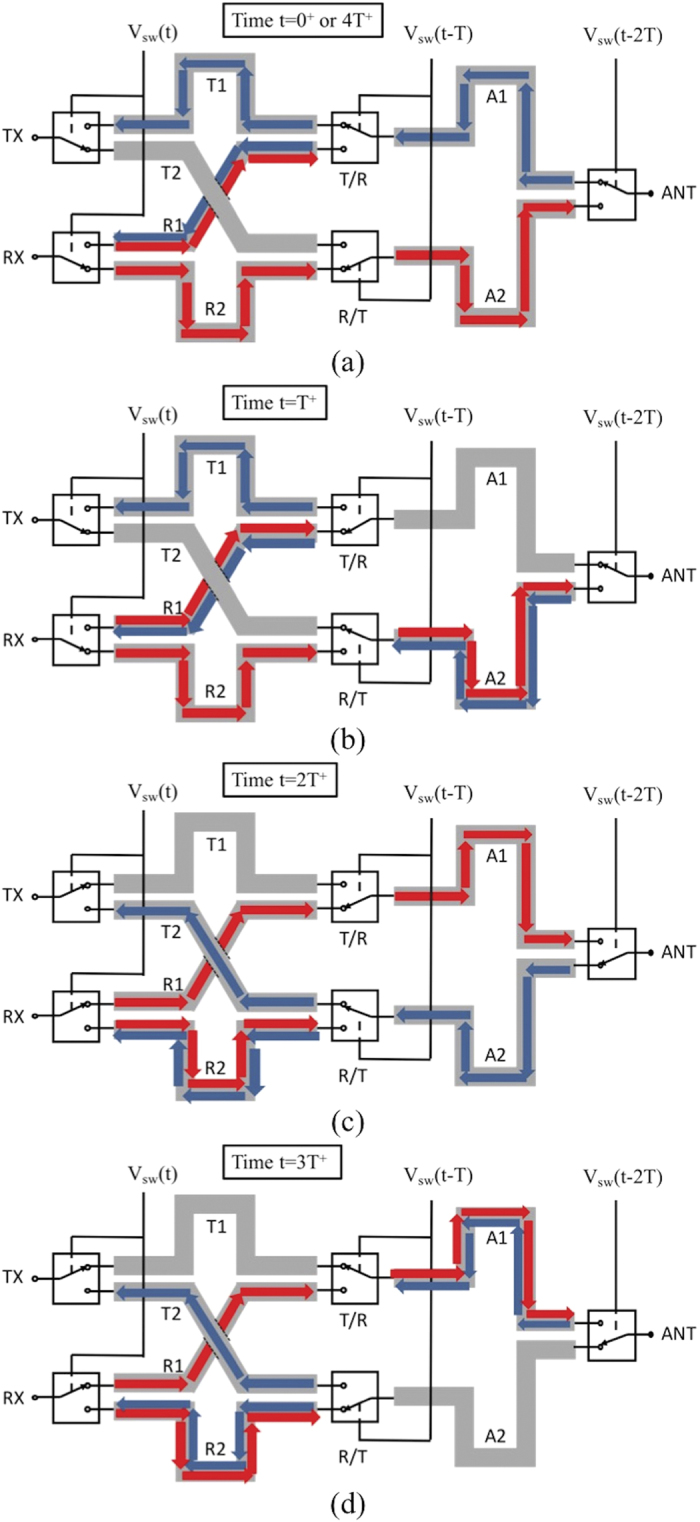
Timing diagram of the waves launched into the RX port during cyclic operations. The transmitted and received signals are represented by the colors red and blue, respectively. Individual figures (**a**) through (**d**) represent the positions of the switches at times 0^+^ to 3*T*^+^ respectively after the device enters into cyclic operations. Each instance in time is with respect to the T/R and R/T switches, and the (+) sign indicates that the switching action has just completed. The time progression shows that the input signals to the RX port continue to propogate to the TX port based upon the switching sequence identical to that in Fig. 2. Note that progressing time to 4*T*^+^ causes the signals to return to their original positions in (**a**).

In Fig. 3a, the wave has propagated on the track of R1 for a time of *T*. Complete reflection is expected as it reaches to the end of R1 that is switched off by the T/R switch. In Fig. 3b, it shows that the wave reflected back by the T/R switch has propagated back to meet the RX switch during a time period of *T* and it will then be reflected again by the RX switch that is turned off and continue its propagation on track R1 toward the antenna direction. In Fig. 3c, the first portion of the wave reaches to the T/R switch again and this time the path is connected through the switch to track A1. At the same time, the second portion of the wave is launched into track R2, starting a process that is similar to what the first portion of wave has gone through. In Fig. 3d, it shows that the first portion of the wave has populated track A1, however, it encounters another open switch, i.e., the ANT switch in the end. In Fig. 3e, the first portion of wave is reflected back to track A1 while the R/T switch connects the second portion of the wave to track A2. In Fig. 3f, the first portion of the wave finally reaches to the TX port with rest of the wave follows. Therefore, it takes a period of 6*T* for the wave launched into the RX port to appear at the TX port. Once that is completed, the device enters into cyclic operations, with the time diagram in Fig. 4 illustrating the wave propagation process at the four switching moments of each cycle. Figure 4 shows that under the identical switching sequence to that in Fig. 2, the waves input into the RX port continue to propagate to the TX port in a cyclic fashion.

## Mathematical Representations

To show the soundness of the SSDL circulator, the following mathematical derivations are performed: The analysis begins by looking at the transmitted signal leaving the TX port, as indicated in Fig. 1. The original transmitted signal is denoted as *TX(t*) and it is split into two pulses that travel respectively along an upper or a lower branch. The upper branch is denoted as “U”, where the first TX pulse travels a transmission line sequence from T1 to A1. The lower branch is denoted as “L” where the second TX pulse travels along the path from T2 to A2. Both pulses finally arrive at the ANT port and combine to form the original transmitted signal. Assuming *V*_*sw*1_(*t*) is the normalized control voltage connected to the first switch of the transmitter, e.g., the demux switch in Fig. 1, the following summation representing its switching behavior:


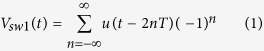


The second and third sets of switches are controlled by the same sequence but at a delay of T and 2T respectively, which are


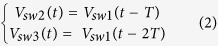


One would then write the two split transmit pulses after the first SPDT switch as the product of the transmit signal and the normalized switch control voltage in the following form,


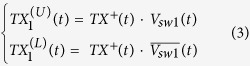


where 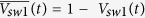 and 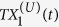 and 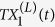 represent the signals just to the right of the TX demux switch that populate T1 and T2 respectively. Both signals are delayed by a time *T* before they arrive at the second set of switches, e.g. the T/R or the R/T switches and output to the paths A1 and A2, respectively. They take the form,


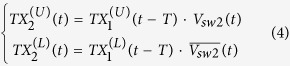


Both signals are further delayed by a time *T* before they arrive at the switch next to the antenna port, e.g, the mux/demux switch, and they are modulated by the mux/demux switch resulting in the following,


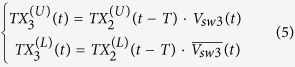


These two signals combine at the antenna port. Substituting (1) through (3) into (4) thus yields the output at the antenna,


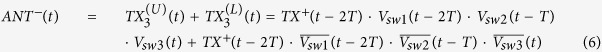


Note that (2) leads to the condition *V*_*sw*3_(*t*) = *V*_*sw*2_(*t*−*T*) = *V*_*sw*1_(*t*−2*T*), and (6) thus becomes


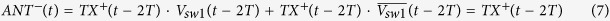


It is evident from (7) that the transmit signal will arrive at the antenna port with merely a delay of 2*T*. It is neither diverted nor altered. For receiving, the signal is received at the antenna port. The mux/demux switch splits the received signal into two pulses that propagate on either the upper or lower branches, in the following form,


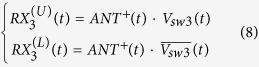


These two pulses take paths A1 and A2, respectively:


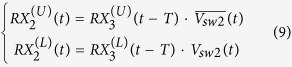


Following the T/R and R/T switches, the two signals arrive at the paths R1 and R2, respectively. Then after a delay time of *T*, both signals arrive at the two inputs of the RX mux switch in the following form,


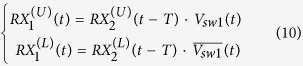


The two signals merge at the RX port with the following expression,


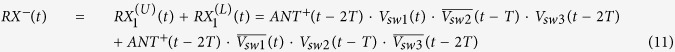


From (1) and (2), it is obvious that 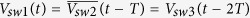. Substituting this into (11) finally yields


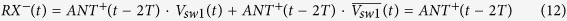


(12) shows that the received signal of the antenna port arrives at the RX port with a time delay of 2*T* as expected.

When the waves are launched into the RX port, a simple mathematical derivation of the complete wave behavior is no longer possible, due to the existence of multiple reflections. However, it is not difficult to derive the following relation with the help of the time diagrams in Figs 3 and 4.


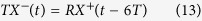


The equations (7), (12) and (13) thus conclude that the operation of SSDL follows that of a three-port circulator except with an asymmetric delay response. For waves traveling from port 3 (RX) to port 1 (TX), a longer delay is expected, which implies a greater loss in practical scenarios.

## Switching Noise Analysis

**Figure 5:**
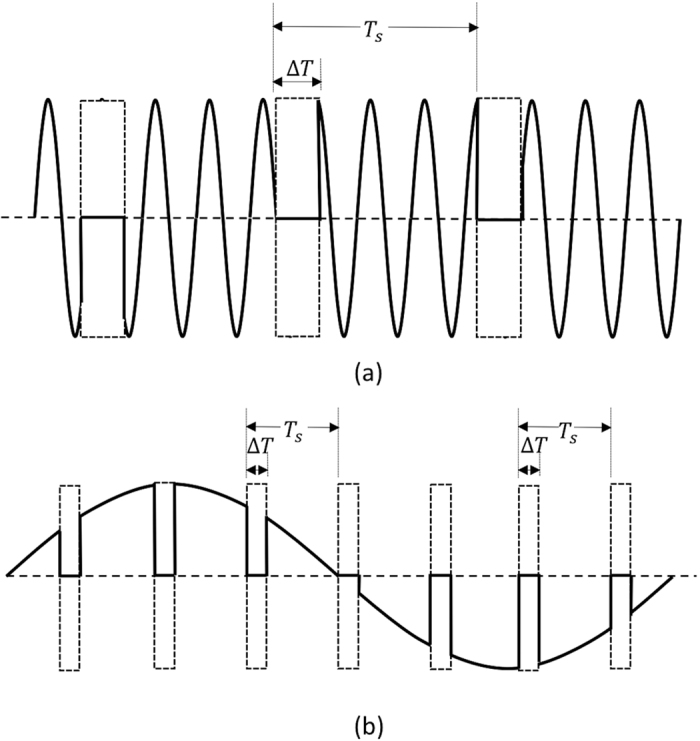
Switching noise represented by periodical rectangular gaps in the signal waveform associated with switching actions. (**a**) the switching rate is much lower than the signal frequency (**b**) the switching rate is much greater than the signal frequency, corresponding to a standard case of oversampling above Nyquist rate.

**Figure 6:**
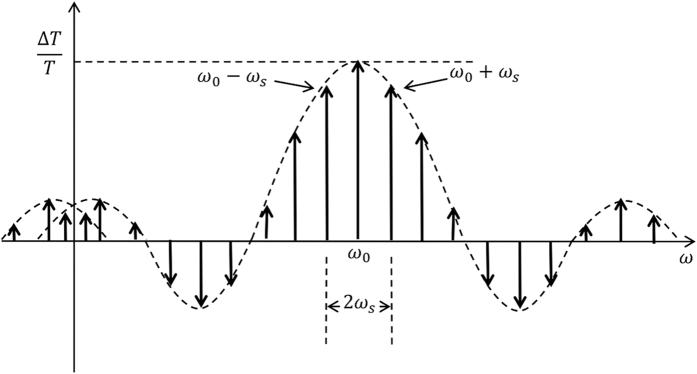
Spectrum of the switching noise when the input signal is a single-tone at the angular frequency ***ω***_0_. The spectrum shows multiple spectral lines spreading according to a SINC function.

The SSDL device operates by splitting the signal waveform in time into two sets of pulses at the input, which are combined later at the output to form the original signal. The process is distortion free if ideal switches with zero transition time are used. In practice, the finite transition time of the switches may cause gaps or spurs in the waveform when the signal energy is lost or discharged during the transition time. The switching noise often behaves as frequency spikes contaminating the signal spectrum. It is thus important to understand the characteristics of such noise. A simplified model of the switching noise is shown in Fig. 5, where periodic gaps are introduced into the signal waveform during the switching actions. The noise is represented by the rectangular gaps appearing at every period of *T*_*s*_, with *T*_*s*_ = 2*T* and the width of the gaps is assumed to be Δ*T*. Figure 5a shows a case when the angular switching rate *ω*_*s*_ = 2*π*/*T*_*s*_ is much lower than the signal angular frequency *ω*_0_ while Fig. 5b displays a case when the switching rate is much greater than the signal frequency. In either case, the switching noise, which is the removed portion in Fig. 5, is expressed by,


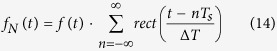


where *f(t)* is the original signal. The spectrum of the switching noise is thus obtained as,


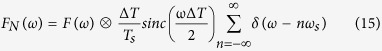


which behaves as the repetition of the original signal spectrum in a period of *ω*_*s*_, following a pattern of SINC function. Assuming a single-tone signal at the angular frequency of *ω*_0_ with an unit amplitude, the spectrum becomes,


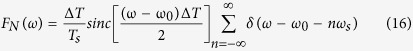


which is represented by the multiple spectral lines plotted in Fig. 6. In general, the noise spectrum scales down in magnitude when the fraction of the transition time versus the switch period reduces. It is observed that the strongest spectral line at *ω*_0_that has a magnitude of Δ*T*/*T*_*s*_ is the lost portion of the original signal that should be counted as the insertion loss rather than noise. Therefore, the strongest noise components are indicated by the two spectral lines at *ω*_0_−*ω*_s_ and *ω*_0_ + *ω*_s_, which has a magnitude of


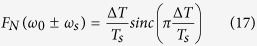


The further noise components in general decay following a SINC function or other spread functions for non-rectangular gap representation of switching noise. In the case *ω*_*s*_ ≪ *ω*_0_, the noise spreading from the negative side of the spectrum may be ignored. It is evident from Fig. 6 that the region between these two spectral lines, i.e. [*ω*_0_ − *ω*_s_*, ω*_0_ + *ω*_*s*_] defines a noise free band of a bandwidth of 2*ω*_*s*_. A bandpass filter with a passband centered at *ω*_0_ may then be utilized to suppress the switching noise at the price of reduced bandwidth of circulator operation. On the other hand, if *ω*_*s*_ ≫ *ω*_0_, one can easily prove that all the noise spectral lines, including those spreading from the negative side, will appear at angular frequencies higher than *ω*_0_. This corresponds to the standard case of sampling above the Nyquist rate, in which case a low pass filter can help to remove all the switching noise.

To prevent switching noise associated with the transmitting signal from entering the receiver, one may also place additional switches in the receiver front-end to block any possible leakage occurring at the proximity of the switching moments. This time gate approach, however, may bring distortions to the received signal in a way similar to the above analysis.

## Circuit Simulation Results

Circuit simulations are carried out in Agilent ADS for a design aiming at on-chip implementation of a circulator operating in the frequency from 500 MHz to 1.5 GHz. The following design parameters are chosen: the switching rate is chosen to be 6 GHz to create an oversampling case. The switching rate corresponds to an 83 ps delay for each transmission line. This corresponds to a straight line length of approximately 10 mm and width of 85 um on a 100 um thick GaN substrate if 50 Ohm microstrip line is used, and the transmission line occupies an actual area of close to 1 mm × 2 mm after meandering. With six such delay lines and 5 transistor switches, the total area of the MMIC should fit well within a chip area of 5 mm × 5 mm.

**Figure 7:**
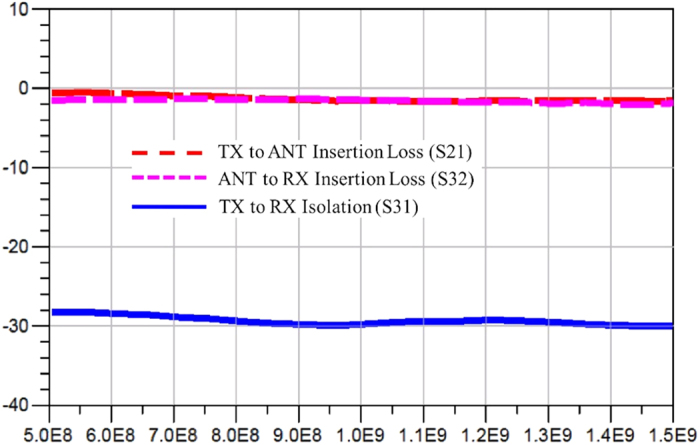
Circuit simulation results for insertion loss (S21 and S32) and isolation (S31) under the denominated usage. The computer simulation results show the insertion loss for the two paths of signal propagation from the TX port to the ANT port and from the ANT port to the RX port. The simulated isolation is between the TX and RX ports. The x-axis displays the frequency range in [Hz]. The y-axis represents the loss and isolation in [dB].

**Figure 8:**
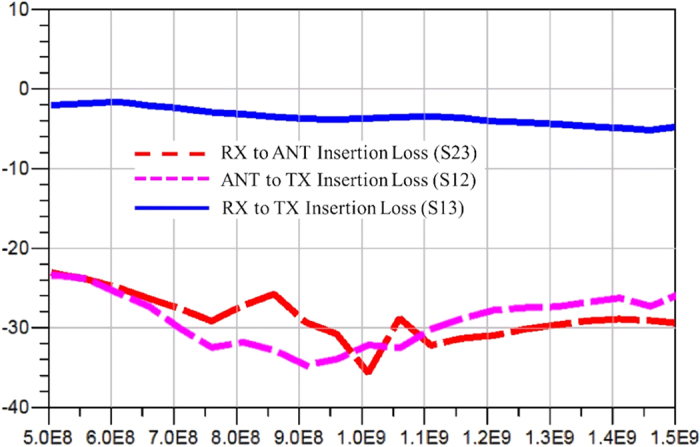
Circuit simulation results for insertion loss (S13) and isolation (S23 and S12) under non-denomiated usage. The computer simulation results show the insertion loss for signals propagating from the RX port to the TX port and isolation for signal propagating from the RX port to the ANT port and from the ANT port to the TX port. The x-axis displays the frequency range in [Hz]. The y-axis represents the loss and isolation in [dB].

Switches are assumed to have a figure of merit similar to what is offered by a typical 0.15 um GaN HEMT switch with an on-resistance of 5 Ohm and off-capacitance of 0.05 pF. The time-domain voltages over all the ports are simulated with ADS transient simulator and the S-parameters in frequency domain is derived by performing Fourier transforms to the time-domain data. When the device is operated as denominated, the simulated insertion losses (S21 and S32) and the isolation (S31) are plotted as functions of frequency shown in Fig. 7, which appear to be very flat curves in general. Approximately 30 dB isolation is obtained with insertion loss less than 1.6 dB for the interested frequency band ranging from 500 MHz to 1.5 GHz. Figure 8 summarized the simulated isolations (S12 and S23) and the insertion loss (S13) when the device is operated in a non-denominated fashion, where worse isolations and greater losses are observed. The insertion loss from the RX port to the TX port has a maximum value of 5 dB due to the multiple reflections and long delays the waves have to experience through that route.

**Figure 9:**
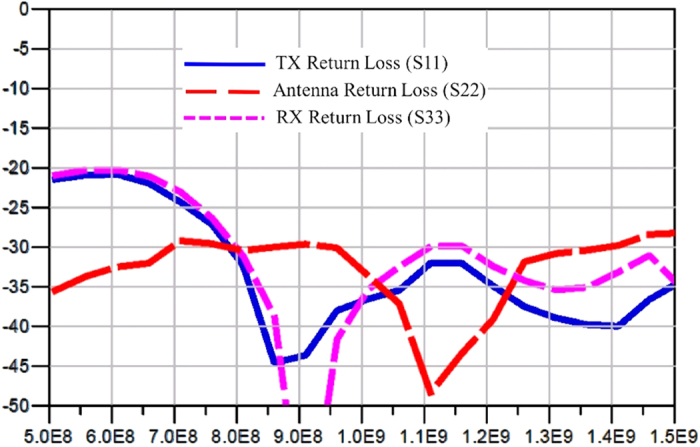
Circuit simulation results for return loss observed at all ports. The findings gathered from the computer simulation show the return loss seen at all of the ports of the proposed circulator. The x-axis displays the frequency range in [Hz]. The y-axis represents the return loss in [dB].

**Figure 10:**
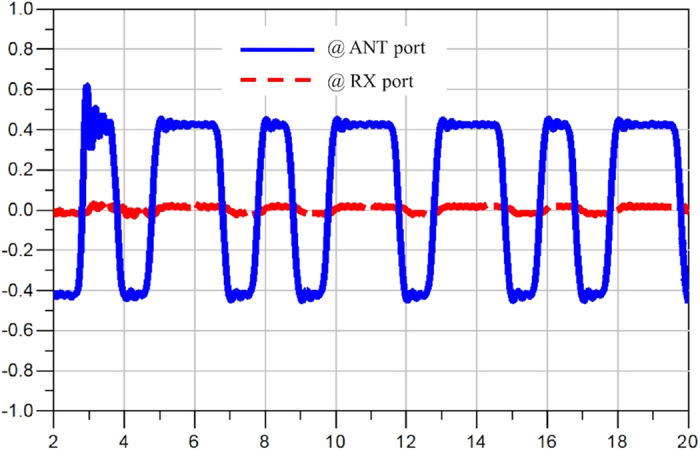
Simulated waveforms appearing at the ANT and RX ports, when waves are launched into the TX port. The blue solid line represents the signal appearing at the ANT port, with a 1Vpp pulses clocked at 1 Gbps with a pattern of 10110101 input at the TX port. The red dashed line is the signal seen at the RX port, with the same excitation provided at the TX port. The x-axis represents time in [ns] and the y-axis represents the amplitude of the signal in [volt].

**Figure 11:**
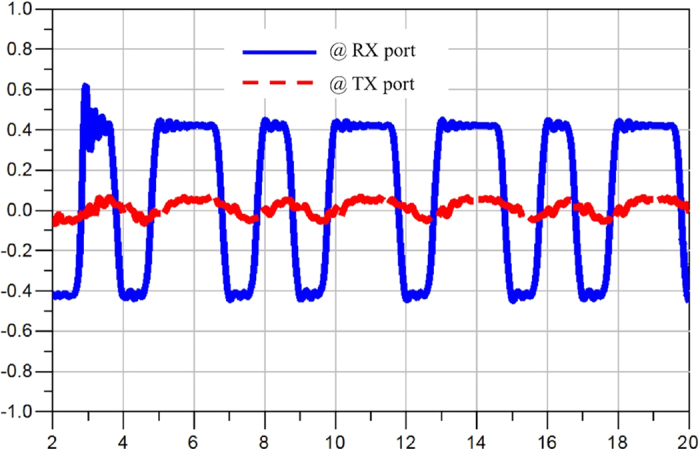
Simulated waveforms appearing at the RX and TX ports, when waves are launched into the ANT port. The blue solid line represents the signal appearing at the RX port, with a 1 Vpp pulses clocked at 1 Gbps with a pattern of 10110101 input at the ANT port. The red dashed line is the signal seen at the TX port, with the same excitation provided at the ANT port. The x-axis represents time in [ns] and the y-axis represents the amplitude of the signal in [volt].

**Figure 12:**
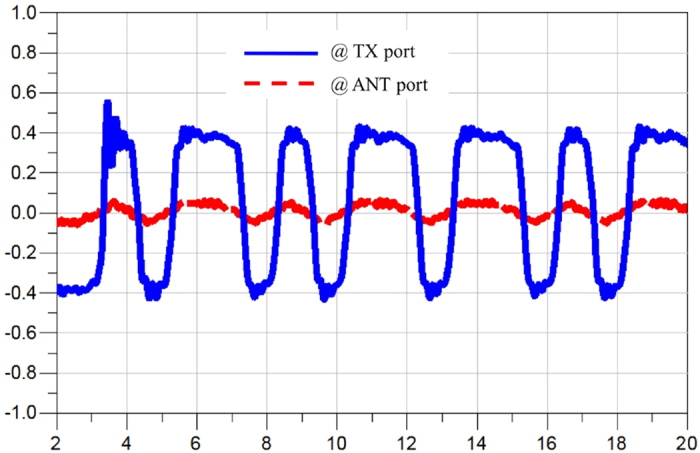
Simulated waveforms appearing at the TX and ANT ports, when waves are launched into the RX port. The blue solid line represents the signal appearing at the TX port, with a 1 Vpp pulses clocked at 1 Gbps with a pattern of 10110101 input at the RX port. The red dashed line is the signal seen at the ANT port, with the same excitation provided at the RX port. The x-axis represents time in [ns] and the y-axis represents the amplitude of the signal in [volt].

Figure 9 shows the simulated return loss from all the ports. At the worst case, the return loss is around 20 dB. Note that a low pass filter was placed at every port to suppress the switching noise, so the return loss is limited by the matching of the filter at each port. Figures 10, 11 and 12 display the waveforms appearing at the other two ports of the SSDL circulator when the TX port, the ANT port and the RX port are respectively excited. The signals are broadband, 1 Vpp pulses clocked at 1 Gbps with a data pattern of 10110101, where “1” is at 0.5 V and “0” is at −0.5 V. Figure 10 indicates that very little leakage is coupled from TX to RX. The leakage from the ANT port to the TX port and from the RX port to the ANT port, are considerably worse from Figs 11 and 12. In addition, the magnitude of the wave is retained for both TX to ANT and ANT to RX paths, while a higher amount of loss and distortion is observed for the RX to TX path as shown in Fig. 12.

## Experimental Methods

**Figure 13:**
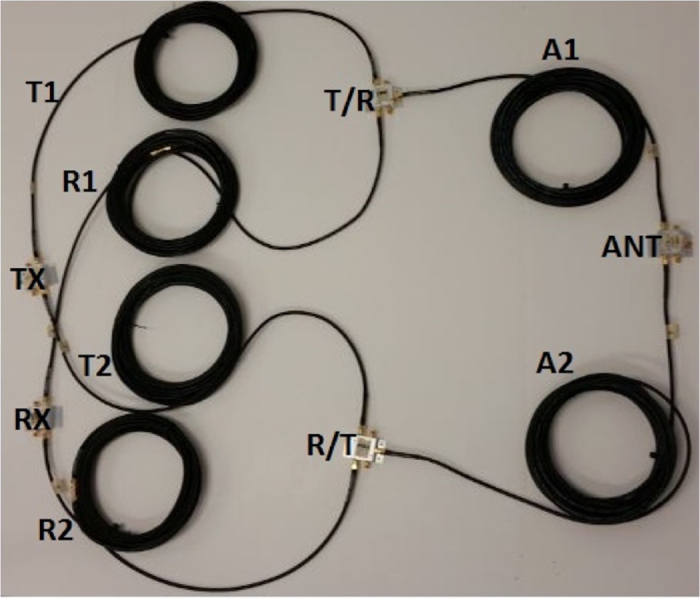
SSDL circulator setup showing the cables and COTS switches. Operation of the experimental setup is from 200 KHz to 200 MHz. This setup allows for simultaneously transmitting and receiving (STAR) of the electromagnetic waves without alterations or reflections to the waveforms.

For proof of concept, an experimental setup with coaxial cables and Commercially Off the Shelf (COTS) switches is used to test the operation of the SSDL device as shown in Fig. 13. It is designed to act as a 1:500 frequency scaled demo to the proposed on-chip circulator. The setup consists of six coaxial cables of equal length with a time delay of *T* of 43 ns. Each individual cable is 30 feet long. The delay time *T* was measured for each of the six transmission lines using a vector network analyzer. The switching action should thus happen at a rate of approximately 24 Msps with each switch turned on or off at the rate of 12 Msps to synchronize with the delay of the cable. Figure 13 also shows five single-pole double-throw (SPDT) switches that are imperative to the simultaneous transmit and receive operation of the device. These switches are available from Mini-circuits (part number ZFSW-2-46). A 10% to 90% transition time of approximately 3 ns and an insertion loss of 1 dB are claimed by the manufacturer. The switches are connected in the SSDL test bed as shown in Fig. 13 among the three radio frequency (RF) ports. The control signals are a series of square waves at 6 MHz with programmed delays, which are generated by the digital outputs of a Sony Tektronix Arbitrary Waveform Generator (part number AWG520).

## Experimental Results

**Figure 14:**
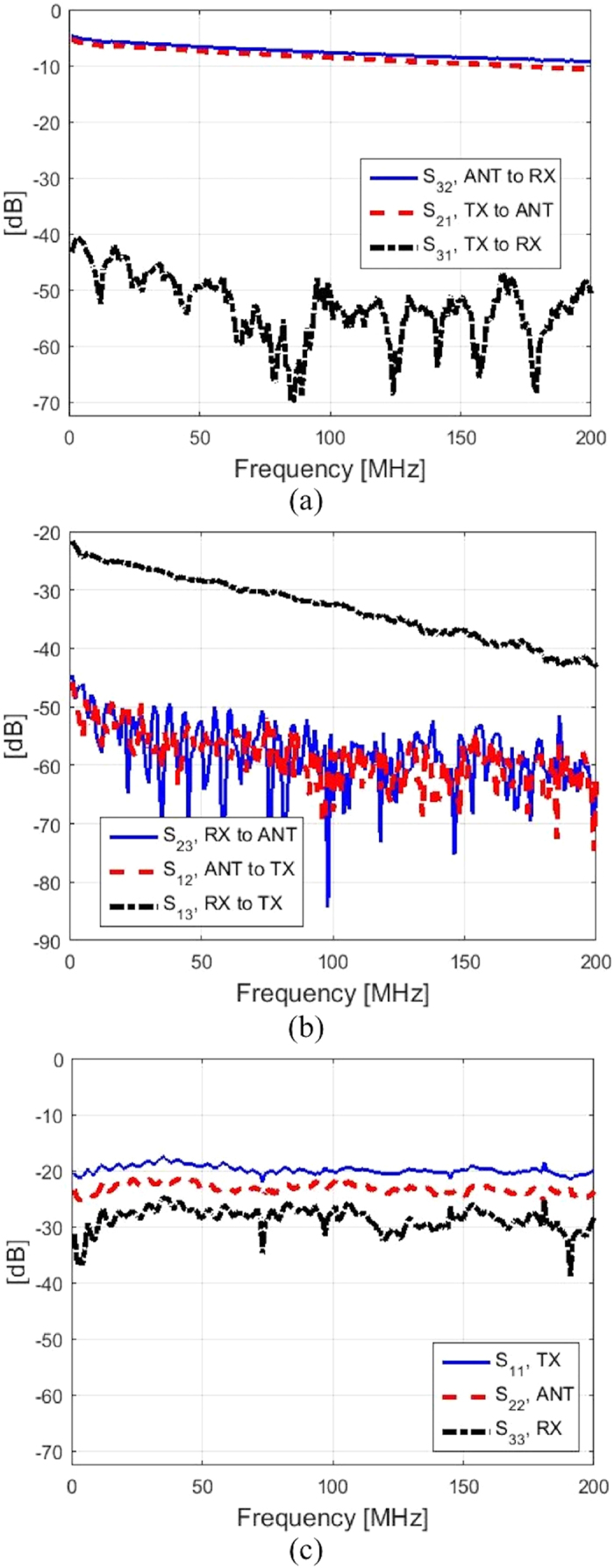
Measured S-parameters for the SSDL circulator using COTS components. (**a**) Measured insertion loss from TX to ANT and from ANT to RX, in contrast to the isolation from TX to RX. (**b**) Measured insertion loss from RX to TX and isolation from ANT to TX and RX to ANT. (**c**) Return loss observed at all of the circulator ports.

S-parameter results are collected from this circulator. The insertion losses, isolation, and port matching parameters were measured using a network analyzer. With the TX, ANT, and RX ports defined as ports 1, 2, and 3, respectively, figure 14 represents the S-parameter results captured. In this case, the start and stop frequencies of the vector network analyzer are set to 200 KHz and 200 MHz, respectively. Figure 14a represents the insertion losses and isolation measured for the circulator. The insertion loss is measured in two ways, e.g. one with S21 being measured from the TX port to the ANT port and the RX port matched with 50 Ohm load, and the second with S32 being measured from the ANT port to the RX port with the TX port matched with 50 Ohm. In both scenarios, the insertion losses range from 5 dB to 10 dB from 200 KHz to 200 MHz, respectively.

It should be noted that the insertion loss of each switch in its stationary mode is approximately 1 dB. At 200 MHz, the loss of each assembled RF cable is approximately 1.5 dB. As the signal propagates from the ANT to RX or TX to ANT path, it passes through two cables and 3 switches. Therefore up to 6 dB insertion loss can then be attributed to the stationary loss of the setup and the rest of the insertion loss can be considered as the dynamic switching loss. It should also be noted that the control voltage outputs from the AWG were below the voltage required that fully turns the switches on and off. The dynamic switching loss can be attributed to this factor as well as the finite transition time of the switches.

To measure the isolation, the forward and reverse ports of the vector network analyzer are attached to the TX and RX ports, respectively. The ANT port is terminated with a matched 50 ohm load, and the isolation loss is measured as S31. The isolation measured between the TX and RX ports is exceptional with approximately 40 dB at the worst case, which occurs around at the frequency of 200 KHz. Figure 14b shows S-parameters for the reverse paths (S23 and S12) which appear to be absorptive in both cases. In particular, the measured S13 shows the insertion loss ranging from 20 to 40 dB, which is significantly greater than the other two forward paths (S21 and S32). It possesses a sharp contrast to the common through behavior in a conventional circulator. This is caused by the multiple reflections and long propagating paths of this path as indicated in the previous analysis. Fortunately a low loss S13 is not required in the primary application of the SSDL circulator, which is to provide full duplex between the transmitting and receiving paths. Figure 14c relates the port matching for all three circulator ports. The measured data shows that all three ports are well matched to the system characteristic impedance of 50 ohms. These results validate that the SSDL circulator demonstrates non-reciprocal behavior from 200 KHz to 200 MHz. In fact, similar non-reciprocal behavior is also observed at 20 KHz, which is the lowest frequency of the vector network analyzer. This shows that indeed there is no lower frequency limit of the circulator’s operation.

**Figure 15:**
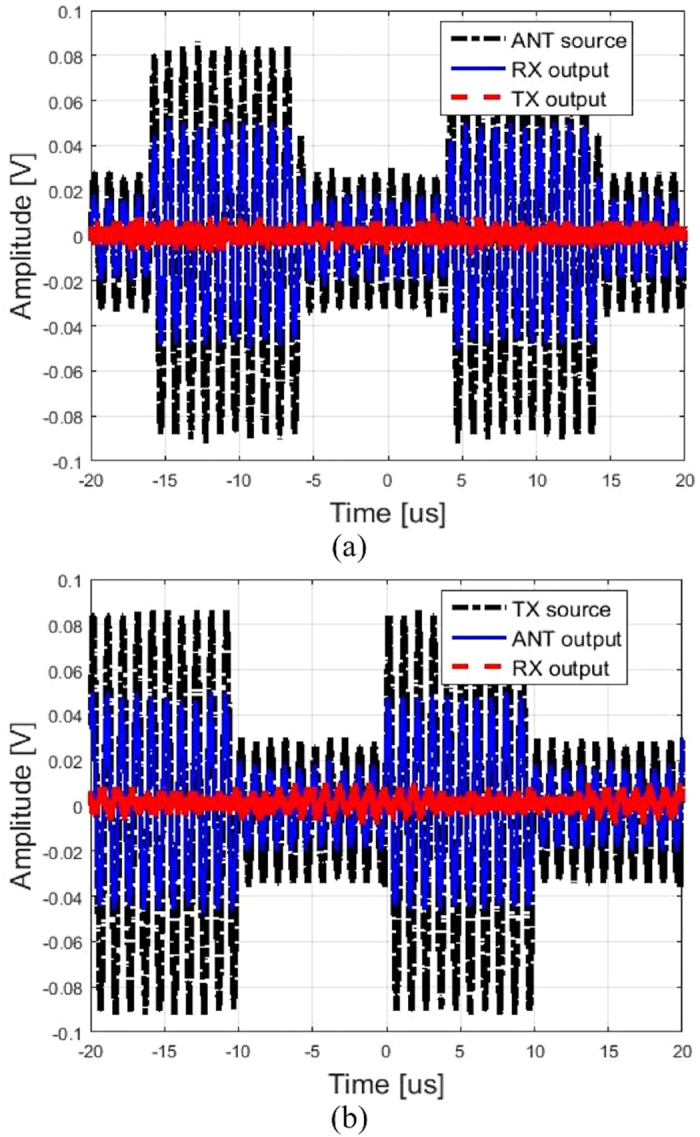
Captured oscilloscope outputs using a modulated input signal. (**a**) The source signal sent from the ANT port is displayed along with the output signals captured at the TX and RX ports. (**b**) The source signal launched from the TX port is shown along with the output waveforms captured at the ANT and RX ports.

To demonstrate its ultra-wide band behavior, the SSDL is also tested with modulated waveforms. Figure 15 showcases the time-domain waveforms from the circulator outputs captured by a Tektronix Digital Phosphor Oscilloscope (part number TDS7404). The input signal is a 1 MHz RF sinusoid modulated by a 50 KHz rectangular wave, supplied by a Tektronix Arbitrary Function Generator (part number AFG3021). To successfully capture and identify the input and output signals without being interfered with by the switching noise, filtering was applied to the outputs of the ANT and RX ports. A Mini-Circuits low pass filter that has a cutoff frequency of 1.9 MHz was attached to each port. In Fig. 15a, the 1 MHz modulated input signal is injected into the low pass filter, which is attached to the ANT port. The RX port output is monitored on the oscilloscope along with the TX port output. As can be seen in Fig. 7a, the input signal is successfully recovered at the RX port. The loss in signal amplitude is the result of dynamic switching and stationary switch/cable losses. Also, very little signal level appears at the TX port, which demonstrates good isolation. For Fig. 15b, the 1 MHz modulated sine wave is supplied to the TX port. The ANT and RX ports are monitored on the oscilloscope. Figure 15b demonstrates the successful transmission of the input signal to the ANT port. The loss due to dynamic switching and stationary switches/cables is again visible, which shows up as the difference between the magnitudes of the ANT output and the TX source. In this case, very little signal level is seen at the RX port, which again demonstrates good isolation.

## Discussion

It is worth noting that there is no absolute requirement of either switching time or delay length enforced by the SSDL operation except the synchronization between the two. The operation of SSDL is independent of frequency theoretically. An ultra-wide band SSDL circulator can be implemented with almost any standard integrated circuit technology including both III-V or silicon based technology, such as GaAs, InP, GaN or CMOS and SiGe. In practical scenarios, switching noise may be of a concern, particularly when the switching frequency is close to the operating frequency. It is thus desired to set the switch frequency to be much higher (or much lower) than the signal frequency for noise sensitive RF applications including STAR or full-duplex communication systems. The spectrum of the switching spurious will be shaped toward the higher end of the spectrum and will not impact the signal band if the switching rate is much higher than the signal frequency. A higher switching rate implies that a shorter delay line can be used, which may be more suitable for on-chip integration.

Due to the passive nature of SSDL, the only power consumption of the device is the power dissipated on turning the switches on and off. In general, transistor switches with smaller gate capacitances will incur less power dissipation, and for this reason high-speed switches are desired. High-speed switches can also help to minimize the loss of the signal and the interference of spurious noise caused by the dynamic switching actions. In addition to the switching speed requirement, the linearity of switches may become an issue for high power operations. Typical SPDT switches on GaAs MMIC can handle up to 1 Watt of RF power without experiencing a significant power compression while switches build with high breakdown voltage process such as those on GaN MMIC may handle more than 10 Watts of RF power.

For low power operations, it is also possible for SSDL to operate with a switching rate much lower than the signal frequency. The switching noise in this case, will appear at the proximity of the signal frequency as shown in Fig. 6. A longer delay, however, is required to synchronize with the slower switching speed. For example, one may combine the long delay of surface acoustic wave (SAW) delay lines and the high speed of semiconductor switches to develop SSDL devices with extremely low power consumption. For optical applications, switching at a rate much lower than the signal frequency may be the only possible operation manner. As electro-optical switches with switching speed in a few nanoseconds have been reported^22^,^23^, they can potentially be used to develop SSDL devices on a silicon optical chip for optical frequencies.

## Additional Information

**How to cite this article:** Biedka, M. M. *et al*. Ultra-Wide Band Non-reciprocity through Sequentially-Switched Delay Lines. *Sci. Rep.* **7**, 40014; doi: 10.1038/srep40014 (2017).

**Publisher's note:** Springer Nature remains neutral with regard to jurisdictional claims in published maps and institutional affiliations.
